# Developmental brain structural atypicalities in autism: a voxel-based morphometry analysis

**DOI:** 10.1186/s13034-022-00443-4

**Published:** 2022-01-31

**Authors:** Hui Wang, Zeng-Hui Ma, Ling-Zi Xu, Liu Yang, Zhao-Zheng Ji, Xin-Zhou Tang, Jing-Ran Liu, Xue Li, Qing-Jiu Cao, Jing Liu

**Affiliations:** Peking University Sixth Hospital, Peking University Institute of Mental Health, NHC Key Laboratory of Mental Health (Peking University), National Clinical Research Center for Mental Disorders (Peking University Sixth Hospital), 51 Huayuan Road, Haidian District, Beijing, 100191 China

**Keywords:** Autism, sMRI, Voxel-based morphometry, Brain structural atypicalities

## Abstract

**Background:**

Structural magnetic resonance imaging (sMRI) studies have shown atypicalities in structural brain changes in individuals with autism spectrum disorder (ASD), while a noticeable discrepancy in their results indicates the necessity of conducting further researches.

**Methods:**

The current study investigated the atypical structural brain features of autistic individuals who aged 6–30 years old. A total of 52 autistic individuals and 50 age-, gender-, and intelligence quotient (IQ)-matched typically developing (TD) individuals were included in this study, and were assigned into three based cohorts: childhood (6–12 years old), adolescence (13–18 years old), and adulthood (19–30 years old). Analyses of whole-brain volume and voxel-based morphometry (VBM) on the sMRI data were conducted.

**Results:**

No significant difference was found in the volumes of whole-brain, gray matter, and white matter between the autism and TD groups in the three age-based cohorts. For VBM analyses, the volumes of gray matter in the right superior temporal gyrus and right inferior parietal lobule in the autism group (6–12 years old) were smaller than those in the TD group; the gray matter volume in the left inferior parietal lobule in the autism group (13–18 years old) was larger than that in the TD group; the gray matter volume in the right middle occipital gyrus in the autism group (19–30 years old) was larger than that in the TD group, and the gray matter volume in the left posterior cingulate gyrus in the autism group was smaller than that in the TD group.

**Conclusion:**

Autistic individuals showed different atypical regional gray matter volumetric changes in childhood, adolescence, and adulthood compared to their TD peers, indicating that it is essential to consider developmental stages of the brain when exploring brain structural atypicalities in autism.

**Supplementary Information:**

The online version contains supplementary material available at 10.1186/s13034-022-00443-4.

## Background

Autism spectrum disorder (ASD) is a neurodevelopmental disorder that appears before the age of 3 years old. Its main clinical manifestations are social communication and communication impairments, restricted and repetitive behaviors, and limited interests [1]. The prevalence of ASD is increasing year-by-year, and it was reported by the Centers for Disease Control and Prevention that the prevalence of ASD was as high as 1/44, seriously impairing the social functions of patients and imposing a heavy burden on their families and society [2]. Previous studies have suggested that the etiology of ASD might be highly related to the interactions between genetic and environmental factors, while its pathological mechanism has still remained elusive [3, 4]. The brain structural developmental atypicalities caused by genetic and environmental factors might be largely involved in the neural mechanisms of ASD, as reported in previous studies [5, 6].

Structural magnetic resonance imaging (sMRI) studies have suggested the accelerated growth of the brain in individuals with ASD shortly after birth, which led to the increase of head circumference and the whole-brain volume [7, 8]. At the age of 2–4 years old, the whole brain volume of individuals with ASD was enlarged by about 10% [9, 10]. The increase in the whole-brain volume of ASD individuals aging 1–2 years old may involve almost all brain regions [11]. Regarding the whole-brain volume study of ASD in childhood, adolescence, and early adulthood, results of the researches were mostly inconsistent [5, 12, 13]. For instance, Aylward Minshew et al. [12] showed that the whole-brain volume of autistic children aging under 12 years old increased by about 5%, whereas the whole-brain volume of autistic adolescents and adults showed no difference compared with typically developing (TD) children. However, another study reported that the total brain volume was enlarged by 5–7% during adolescence [5]. Moreover, Riddle Cascio et al. [14] used voxel-based morphometry (VBM) to analyze sMRI data, a method that examines structural changes of the brain at the millimeter range, and found that the total brain and grey matter (GM) volumes were enlarged by approximately 1–2% in ASD, however, the effect reached statistical significance on only the cohort of all subjects, rather than on childhood, adolescence, and adulthood cohorts. Regarding the total brain GM volume in ASD, Freitag Luders et al. [13] found that the GM volume of adolescents in the ASD group increased compared with the TD group. Mitelman Bralet et al. [15] demonstrated that the GM volume in autistic adults also increased compared with TD adults who aged 21–34 years old. Some studies also reported the correlation of autistic symptoms with the structural atypicalities in ASD individuals. Mitchell et al. suggested that the reduction in the volume of the dorsolateral prefrontal cortex in ASD individuals was correlated with social and communication scores of Autism Diagnostic Observation Schedule (ADOS) [16]. Hollander et al. reported that the increase in the volume of the right caudate nucleus in ASD adults was positively correlated with the Restricted and Repetitive Behaviors (RRB) score of Autism Diagnostic Interview (ADI) [17].

The results of the regional specificity of structural brain abnormalities in ASD in childhood, adolescence, and early adulthood were also inconsistent [5, 11, 12]. Previous studies have suggested that the increase of total brain volume after birth in ASD individuals may involve the increase in the volume of almost all brain regions [11], including the frontal lobe [18, 19], temporal lobe [9], occipital lobe [20], cerebellum [21], amygdala [22], or the increased volume of some brain regions and decreased volume of other brain regions [23]. A previous research demonstrated that brain overgrowth in early ASD mainly involves cortical thickening [20]. The results of the regional specificity of structural brain abnormalities in autistic children, adolescents, and adults were widely reported, including frontal lobe [24, 25], temporal lobe [26], parietal lobe [27], occipital lobe [27], amygdala [28], caudate nucleus [29], hippocampus [24], thalamus [5], and cerebellum [30].

The inconsistencies in the results of sMRI studies of ASD might be correlated with several factors: first, the participants’ range of age who were included in those studies was inconsistent, including childhood, adolescence, and adulthood cohorts [20]. These inconsistencies might cause difficulties in summarizing atypical structural brain areas at different developmental stages. Second, these inconsistencies in brain structure atypicalities in ASD were also related to participants’ gender and intelligence quotient (IQ) [31]. Third, in a number of those studies, the small sample size may also contribute to the inconsistencies in the results.

The present cross-sectional study included sMRI data of autistic individuals and TD individuals who aged 6–30 years old to explore the age-related differences of whole-brain volume and the GM volume between the two groups. Different analyses were conducted on the three age-based cohorts: late childhood (6–12 years old), adolescence (13–18 years old), and adulthood (19–30 years old). We predicted that probably no significant group-level brain structural atypicalities would be found between the two groups in the cohort of all subjects (i.e., 6–30 years old). However, the whole-brain volume and/or regional specificity of structural brain abnormalities in childhood, adolescence, and adulthood cohorts could be identified in the autism group compared with TD group.

## Methods

### Participants

A total of 52 high-functioning autistic individuals who aged 6–30 years old and met the Diagnostic and Statistical Manual-IV (DSM-IV) criteria for autism were recruited from the outpatient clinic of the Peking University Sixth Hospital (Beijing, China) between March 2013 and January 2017. The diagnosis was performed by two deputy chief physicians or chief physicians using the DSM-IV criteria. Besides, 50 TD individuals who aged 6–30 years old were enrolled from January 2016 to January 2017. All participants were right-handed and had an IQ greater than 70 measured with either the Chinese-Wechsler Intelligence Scale for Children (C-WISC) or the Wechsler Adult Intelligence Scale-Revised in China (WAIS-RC) [32, 33]. Participants were excluded if they had mental disorders (other than autism), suffered from severe physical diseases, neurological diseases and brain trauma, with a history of consumption of psychotropic drugs, being unable to cooperate in examinations, or had metal implants (e.g., non-removable dentures). All participants were divided into three age-based cohorts as follows: childhood: 6–12 years old (autism: n = 24; TD: n = 19); adolescence: 13–18 years old (autism: n = 18; TD: n = 18); and adulthood: 19–30 years old (autism: n = 10; TD: n = 13). Age, gender, and IQ were matched for autism and TD groups in each age-based group (refer to Table 1 for demographic data). This study was approved by the Ethics Committee of the Sixth Hospital of Peking University. Children and their guardians, as well as adult subjects understood the content and objective of the study and agreed to participate in the study. All children’s guardians and adult subjects signed the written informed consent forms.

**Table 1:** Demographic information

Group	autism (n = 52)	TD (n = 50)	*t/x*^*2*^	*P*
	Mean ± SD	Mean ± SD		
6–12 years	n = 24	n = 19		
Age	9 ± 2.0	9 ± 1.7	− 0.278	0.783
Full IQ	102.4 ± 19.6	109.7 ± 12.9	− 1.479	0.147
Gender (male/female)	21/3	11/8	3.451	0.063
13–18 years	n = 18	n = 18		
Age	14.1 ± 1.2	14.7 ± 2.1	− 1.051	0.301
Full IQ	108.7 ± 13.1	116.5 ± 10.0	− 1.996	0.054
Gender (male/female)	15/3	14/4	0.000	1.000
19–30 years	n = 10	n = 13		
Age	21.9 ± 3.0	22.6 ± 3.8	− 0.487	0.631
Full IQ	115.1 ± 17.9	121.5 ± 5.3	− 1.098	0.297
Gender (male/female)	9/1	12/1	0.000	1.000

### MRI data collection

All MRI data were collected by the GE Discovery 750 3.0T magnetic resonance scanner (GE Healthcare, Chicago, IL, USA) in the Peking University Third Hospital using 8-channel phased array head coil. Subjects were placed in supine position and fixed with foam pad during scanning. Besides, three-dimensional (3D) T1 SPGR sequence sagittal scanning was carried out with the following parameters: repetition time (TR) = 4.78 ms, echo time (TE) = 2.02 ms, flip angle = 15°, field of view (FOV) = 24 mm × 24 mm, matrix size = 240 × 240, slice thickness = 1.0 mm, voxel size = 1.0 mm × 1.0 mm × 1.0 mm; 166 slices of images were collected from the whole-brain.

### MRI data processing

All MRI data were processed using MATLAB R2009a software. Image preprocessing was conducted based on the VBM8-DARTEL toolbox (http://dbm.neuro.uni-je-na.de/vbm) in SPM8 software (http://www.fil.ion.ucl.ac.uk/spm/software/spm8). Each time, only one age-based group of autism and TD groups was processed. The three age-based cohorts were processed as follows: (a) AC-PC correction for MR images; (b) anatomical segmentation of T1-weighted structural images using segmentation template of ‘New Segment’ to extract the original image and volumes of GM, white matter (WM), and cerebrospinal fluid (CBF); (c) using DARTEL toolbox to average each part of the image, to achieve initial registration template, followed by matching the images and templates according to WM, GM, and CBF volumes to obtain the re-averaged images and to generate the optimal template of DARTEL through six iterations; (d) utilization of nonlinear transformation to match the initial segmentation image to the optimal template of DARTEL; (e) the deformable field obtained by the DARTEL was used to register the images at the Montreal Neurological Institute (MNI) platform and to generate volumetric modulated maps. All image positions and individual voxel sizes (1.5 mm × 1.5 mm × 1.5 mm) were aligned to achieve spatial volume comparability; (f) Gauss smoothing of standardized images was performed (full width at half maximum (FWHM) = 6 mm) to improve the signal-to-noise ratio (SNR) of MR images.

### Statistical analysis

For the statistical analysis of whole-brain volume, single variable covariance analysis was employed, with inclusion of whole-brain volume, whole-WM volume, and whole-GM volume as dependent variables, as well as gender, age, and IQ as covariates.

To perform VBM analysis in SPM8 software, using a generalized linear model, the smoothed gray matter images of autism and TD groups were tested by voxel-based double-sample *t*-test. Age, gender, IQ, and intracranial volume (WM volume + GM volume + CBF volume) were taken as covariates, and the statistical analysis of whole-brain volume was carried out voxel-by-voxel. The Gaussian random field theory was used for multiple comparisons. Threshold of voxel level was set to P < 0.001, and the cluster number of voxels was considered significant if it was more than 50 [34].

## Results

### Whole-brain volume

The comparison of the volumes of whole-brain, whole-GM, and whole-WM between the two groups in distinct developmental cohorts (6–12, 13–18, and 19–30 years old) showed that, there was no significant difference between the autism and TD groups in the three age-based cohorts (all *P* > 0.05) (Additional file 1: Tables S1–S3).

### Regional GM volume in ASD: VBM

**Fig. 1:**
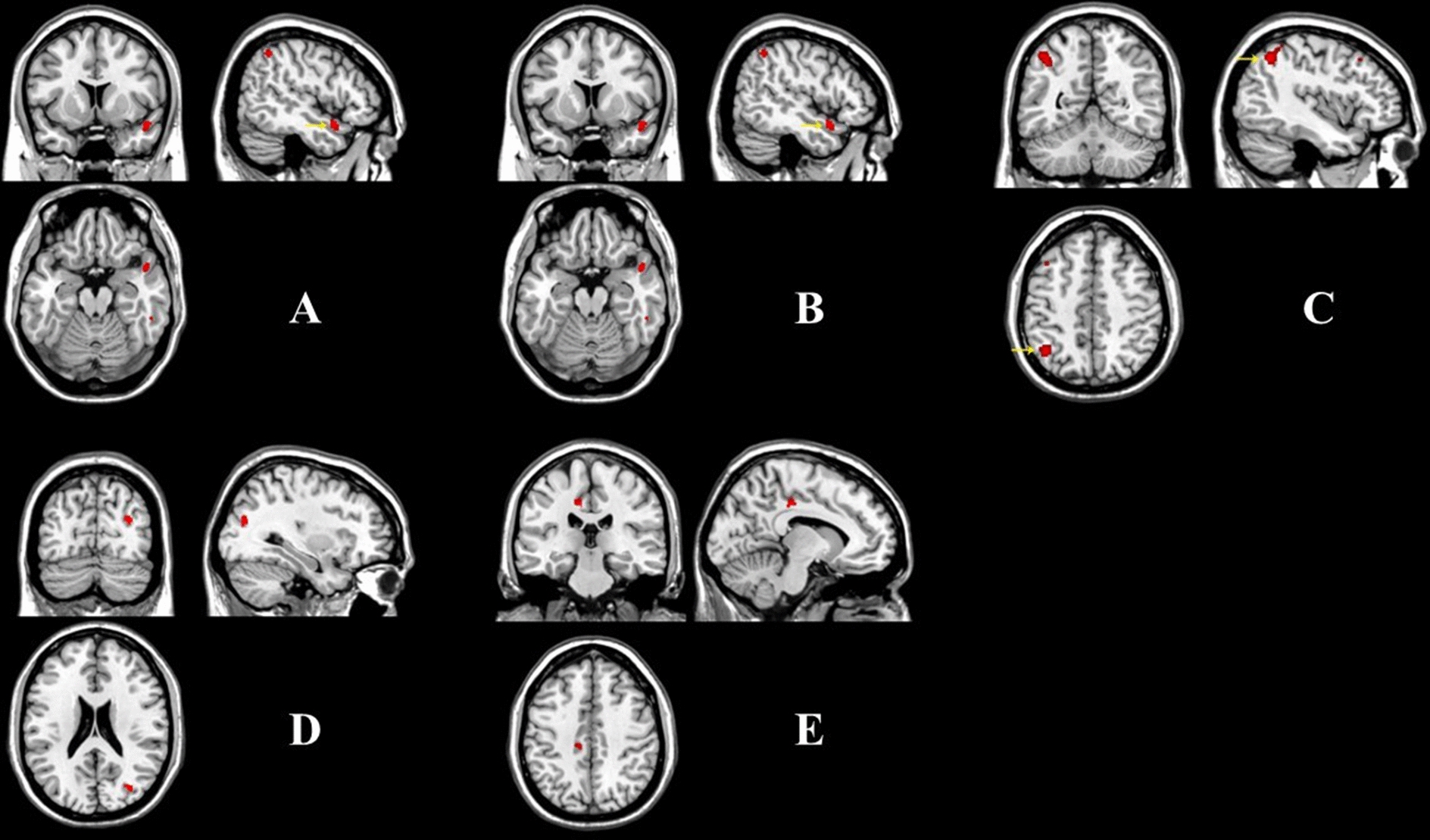
Regions showing significant differences of grey matter volume between ASD and TD groups based on VBM analyses (uncorrected). **A** Right superior temporal gyrus; **B** Right inferior parietal lobule; **C** Left inferior parietal lobule; **D** Right middle occipital gyrus; **E** Left anterior cingulate gyrus

6–12 years old: In the older childhood cohort, two regions showed a significantly smaller GM volume in the autism group than that in the TD group: clusters of right superior temporal gyrus and right inferior parietal lobule (*P* < 0.001, uncorrected, cluster number > 50, voxel size = 3.375). No region was found in the autism group with a greater volume than the TD group (Fig. 1 and Table 2).

**Table 2:** Regions showing significant differences of grey matter volume between ASD and TD groups based on VBM analyses in three age groups (uncorrected)

Regions	BA	Voxel number	Peak MNI			Peak intensity
			X	Y	Z	
6–12 years old						
ASD > TD	No significant results					
TD > ASD						
Right superior temporal gyrus	22	102	39	− 18	− 3	− 4.088
Right inferior parietal lobule	40	90	48	− 51	49.5	
13–18 years old						
ASD > TD						
Left inferior parietal lobule	40	357	− 42	− 57	48	5.966
TD > ASD	No significant results					
19–30 years old						
ASD > TD						
Right middle occipital gyrus	19	86	33	− 75	22.5	4.281
TD > ASD						
Left anterior cingulate gyrus	31	80	− 9	− 27	37.5	− 4.905

Within-cluster peaks were identified based on DPABI (http://rfmri.org/dpabi) and CUI Xu's xjview (http://www.alivelearn.net/xjview/). Cluster size is reported in number of voxels (2 × 2 × 2 mm). *BA* Broca’s area

13–18 years old: In the adolescent cohort, one region showed a significantly greater GM volume in the autism group than that in the TD group: left inferior parietal lobule (*P* < 0.001, uncorrected, cluster number > 50, voxel size = 3.375). No region was found in the autism group with a smaller volume than the TD group (Fig. 1 and Table 2).

19–30 years old: In the young adult cohort, one region showed a significantly larger GM volume in the autism group than that in the TD group: right middle occipital gyrus (*P* < 0.001, uncorrected, cluster number > 50, voxel size = 3.375). One region demonstrated a significantly smaller GM volume in the autism group than that in the TD group: left anterior cingulate gyrus (*P* < 0.001, uncorrected, cluster number > 50, voxel size = 3.375) (Fig. 1 and Table 2).

## Discussion

The current study included 52 autistic individuals and 50 TD individuals, who were matched for age, gender, and IQ. No significant difference was found in the volumes of whole-brain, whole-GM, and whole-WM between the two groups in distinct developmental stages (6–12, 13–18, and 19–30 years old). However, using VBM analyses, different GM regions showed significant differences in volume between the autism and TD groups in different age-based cohorts, involving parietal lobe, occipital lobe, temporal lobe, and cingulate gyrus.

## Whole-brain volume in the autism

The present study compared the whole-brain volume, whole-GM volume, and whole-WM volume of subjects between the autism and TD groups among the three age-based cohorts (6–12, 13–18, and 19–30 years old), and no significant volume-based difference was noted between the two groups in each age-based cohort. The results were consistent with previous studies, which demonstrated that autistic individuals exhibited no significant differences on whole-brain volume, whole-GM volume, and whole-WM volume compared with TD individuals.

Regarding the comparison of whole-brain volume, Jou Minshew et al. [35] recruited 22 autistic children and 22 TD children who aged 8–12 years old, and they found no significant difference in the whole-brain volume between the two groups. Tepest Jacobi et al. [36] enrolled 29 autistic adults and 29 TD adults, and also found no significant difference in whole-brain volume between two groups. Riddle Cascio et al. [14] used sMRI data from a large cohort that included 539 autistic patients and 573 healthy controls, and they found no significant difference in the whole-brain volume between childhood (6–12.6 years old), early adolescence (12.7–16.1 years old), late adolescence (16.2–22 years old), and adult (older than 22 years old) autistic and TD individuals.

Regarding the analysis of whole-brain GM volume, one study recruited 86 autistic individuals and 90 TD individuals who aged 7–29 years old, and the results showed that there was no significant difference in the whole-brain GM volume between the two groups [37]. Another study suggested that the differences in GM volume between autistic and TD adolescents and adults were more reflected in the imbalance of GM volume in local brain regions than in whole-brain GM volume [37].

Regarding the comparison of whole-brain WM volume, Radua Via et al. [38] conducted a meta-analysis of the articles that were recorded in the PubMed database from 2002 to 2010 and concentrated on WM volume of autistic individuals, and they found no significant difference in the whole-brain WM volume between ASD and TD groups.

However, few previous studies also showed that the whole-brain volume of ASD group was larger than that of TD group, which was inconsistent with the results of the present study. This inconsistency could be largely related to different IQ ranges and sample size in the two groups. For instance, Freitag Luders et al. [13] enrolled 15 autistic individuals and 15 TD individuals who aged 14–22 years old, and the results of the IQ test showed significant differences between the two groups, and they also found that the whole-brain volume, whole-brain GM volume, and whole-brain GM volume of the ASD group were all larger than those of the TD group. To better compare the whole-brain volume between autistic and TD individuals, participants with different IQ ranges and larger sample size need to be involved in the future studies.

## Atypical regional GM volume in the autism group in the three age-based cohorts: VBM

### Right superior temporal gyrus

It was found in the current study that the GM volume in the right superior temporal gyrus of 6–12-year-old autism group was smaller than that of the TD group, while there was no significant difference between the two groups in age-based cohorts of 13–18 and 19–30 years old. Previous studies have shown that the superior temporal gyrus was closely associated with language [39], visual function [25], auditory function [40], and social cognition [41]. Social cognition refers to the processing of facial expression, eye gaze, physical movement, and other information by individuals in social communication. Its main objective is to recognize and understand individuals’ mental status [42]. The posterior part of superior temporal gyrus, which involves advanced cortical integration function, integrates sensory information and limbic system information, is the core cortical area of social brain [43]. A previous research [26] recruited 21 autistic children and 12 healthy controls who aged 7–11 years old, and it was revealed that the density of GM in superior temporal sulcus of autistic individuals decreased compared with TD individuals, which supported the results of the present study. Kates Mostofsky et al. [44] analyzed brain sMRI data of five 7-year-old autistic twins and found that the volumes of their superior temporal gyrus were smaller than those of TD children. From twin studies, structural atypicalities of superior temporal gyrus were noted to be correlated with heredity in ASD.

Compared with left superior temporal gyrus, right superior temporal gyrus has more important functional significance for autistic individuals. Previous studies have found that the right superior temporal gyrus in ASD individuals played a dual-role in language and social cognition. Boddaert Belin et al. [45] found that the activation of left superior temporal gyrus was more obvious than that of the right side in healthy controls when recognizing and understanding speech, while the activation of the right superior temporal gyrus was more obvious in autistic adults. A previous research [46] found that delayed development of the integrated function of design action and language in autistic children was related to the development of right superior temporal gyrus. The above-mentioned studies may indicate that the right superior temporal gyrus atypicalities are closely related to autistic symptoms, and have important pathological significance for autistic individuals.

In the present study, no significant difference was detected in the GM volume of the right superior temporal gyrus between the autism and TD groups in the cohorts of 13–18 and 19–30 years old. The atypicality of GM volume in the right superior temporal gyrus was considered age-dependent, as shown in numerous previous studies. A prospective study of GM development included 100 autistic individuals and 117 TD individuals who aged 3–34 years old, and they found that the GM volume in temporal lobe decreased with the increase of age in both groups. The developmental trajectory analyses showed that the GM volume in temporal lobe of ASD group was smaller than that of TD group before the age of 14 years old, while there was no significant difference between the two groups after 14 years old [47]. Dickstein Pescosolido et al. [48] conducted a meta-analysis of the task-based MRI study of ASD (535 autistic children and 604 autistic adults), and found that during social tasks, the right superior temporal gyrus of autistic children was less activated than that of autistic adults. Therefore, it is essential to include longitudinal data to assess the volume of this brain area in ASD individuals at different developmental stages, which is the gold standard of developmental studies.

In addition, a number of scholars have found that changes in cell volume in superior temporal gyrus were associated with ASD [41], which further explained the pathology of the superior temporal gyrus in ASD. A previous research [49] found that the superior temporal gyrus of autistic individuals was associated with the increased transcription levels of several immune system-related genes with noticeable variations, which was noted to be associated with the characteristic innate immune response of neurodevelopmental diseases. It is therefore highly advantageous to further identify susceptible genes and pathological mechanism.

Although the results of the present study are mainly consistent with most previous studies, there are still some inconsistencies. For instance, one study enrolled 18 autistic individuals and 19 TD individuals who aged 10–16 years old, and found that the right superior temporal gyrus volume significantly increased in ASD group. The discrepancy could be related to the fact that the mentioned study only compared the right superior temporal gyrus volume without distinguishing WM from GM [41, 50]. Other studies did not find atypical volume of left superior temporal gyrus in autistic individuals, or reported that the left superior temporal gyrus volume decreased in autistic individuals rather than in the right side. The inconsistencies in these results could be related to numerous factors, including methodological differences, differences in participants’ clinical data, etc.

### Inferior parietal lobule

The current study found that the GM volume of the right inferior parietal lobule was smaller in the autism group than that in the TD group in children (6–12 years old), and the GM volume of the left inferior parietal lobule was larger in the autism group than that in the TD group in adolescents (13–18 years old). No significant difference was observed in the GM volume of bilateral inferior parietal lobule between the autism group and TD group in young adults (19–30 years old). The inferior parietal lobule is involved in sensory input, especially visual and spatial localization [51]. It is also a part of human mirror nervous system [52] involving image thinking, imitative action [53], eye contact [54], and semantic processing [55], and was considered as one of the most highly connected hubs in brain [56]. Venkataraman Duncan et al. [57] found that the left inferior parietal lobule of ASD individuals was involved in the formation of social pathological networks. A meta-analysis of fMRI studies revealed that the anterior inferior parietal lobule of ASD individuals was atypically activated during observation and imitation, and mirror neuron dysfunction existed [52]. Several previous studies have shown that the GM volume in the right inferior parietal lobule of autistic children was smaller than that of the TD children, and the reduction of GM volume was positively correlated with the severity of social disorders [58]. Mengotti D’Agostini et al. [27] enrolled 20 autistic children and 22 TD children (4–14 years old), and they found that the GM volume in the left parietal lobule was larger in autistic children than that in TD children. The above-mentioned findings all supported the results of the present study. Piven Arndt et al. [59] enrolled 35 autistic individuals and 36 TD individuals (12–29 years old), and demonstrated that the parietal lobe volume of autistic individuals was enlarged compared with that in TD individuals, which was partly consistent with the results of our study.

The current study found that the left inferior parietal lobule in the autism group was larger in adolescence than that in the TD group, whereas there was no significant difference between autistic adults and TD adults. It is speculated that the GM volume in the left inferior parietal lobule of ASD individuals may gradually decrease from adolescence to adulthood, and similar results have been reported by previous studies. A study on the brain structure of autistic adolescents included 25 autistic individuals and 25 TD individuals (10–18 years old). The results showed that the GM volume in bilateral inferior parietal lobule decreased with the increase of age in ASD group, while it increased with the elevation of age in TD group [58], which was in agreement with the results of the current study. Christian et al. recruited 28 autistic adults and 28 TD adults (20–55 years old), and found that the left inferior parietal lobule of autistic individuals was thinner than that of the TD individuals and tended to decrease with age in ASD individuals [60].

The results of the present study revealed that the atypicalities of bilateral inferior parietal lobules in autistic individuals of different age-based cohorts were different. This could be related to the involvement of the lateralization of brain structure, as well as the influences of the age-dependent factors on the development of inferior parietal lobules in ASD individuals. A previous study reported that the left inferior parietal lobule of autistic adolescents was larger than the right lobule [58]. Another study also found an increase in the left cerebral asymmetry in autistic individuals [61], which supported the results of the current study.

Based on the above-mentioned studies, despite the differences in age and clinical characteristics in those studies examining parietal lobules of autistic individuals or the inconsistency of previous research results, a great number of studies have shown that bilateral inferior parietal lobules of autistic individuals were significantly different in structure and function from those of TD individuals, which fully illustrated the pathological significance of bilateral inferior parietal lobules of ASD individuals. The results of the present study also provided evidence for atypicalities of bilateral inferior parietal lobules of ASD individuals.

### Right middle occipital gyrus

The current study revealed that the GM volume in the right middle occipital gyrus in the autism group (19–30 years old) was larger than that in the TD group, and there was no significant difference between the autism and TD groups in age-based cohorts of 6–12 and 13–18 years old. The results of the current study were partly consistent with those of previous studies. One study included 38 autistic individuals and 46 TD individuals who aged 6–17 years old. No significant difference was found in GM volume of middle occipital lobe [62]. Ecker Marquand et al. [63] enrolled autistic adults and TD adults who aged 20–68 years old and found that the occipital lobe cortex of autistic individuals was thicker than that of TD individuals. The above-mentioned results were consistent with the findings of the present study. Piven Arndt et al. [59] enrolled 35 autistic individuals and 36 TD individuals (12–29 years old, 18 years old on average). It was found that the occipital lobe volume in autistic group was enlarged compared with the TD group, which partly supported the results of the present study. However, there have been inconsistencies in the results of previous reports and the current study. A meta-analysis of GM atypicalities examining autistic individuals who aged 6–14 years old, and it was found that the GM volumes in the left anterior occipital gyrus and left inferior occipital gyrus of autistic individuals were enlarged [64] compared with TD individuals, while there was no significant difference in occipital lobe volume between 6 and 18-year-old autism group and control group in the current study. Another study enrolled autistic individuals with an average age of 26 years old and TD individuals, and they found that the GM volume in occipital lobe of ASD group was smaller [65] compared with TD group. Some studies found that the size of occipital lobe was correlated to IQ and the severity of ASD. Therefore, the reasons for the inconsistencies in those results could be related to a variety of factors, including sample size, age, IQ, severity of symptoms, and lateralization anormaly of occipital lobe. Despite inconsistencies in the results of previous studies, one meta-analysis on GM atypicalities in ASD revealed that most studies showed structural atypicalities in the occipital lobe of autistic individuals [20].

The occipital lobe is responsible for visual spatial information processing, as well as the processing of body language and emotional regulation [66]. Previous fMRI studies have shown that autistic individuals relied more on occipital primary visual function to encode external information in social and non-social tasks, while TD individuals relied more on language [67]. Autistic individuals atypically activated the occipital gyrus rather than the traditional spindle facial area during facial processing tasks [28]. Besides, a previous fMRI study revealed that there were atypical developmental patterns in the middle occipital gyrus of autistic children and adolescents compared with TD individuals [68]. Besides, the atypical development of the occipital lobe could also be related to the altered gene expression or neurometabolity. Ginsberg Rubin et al. [69] found atypical gene expression in occipital lobe of autistic adults, including mitochondrial oxidative phosphorylation and down-regulation of protein translation genes. Levitt O’Neill et al. [70] found atypical neurometabolites in the occipital cortex of autistic individuals who aged 5–16 years old using proton magnetic resonance spectroscopy. Therefore, molecular and imaging studies suggested that occipital lobe and middle occipital gyrus played an important role in the pathogenesis of ASD. The results of the present study provided more reliable evidence for occipital lobe atypicalities in ASD.

### Left posterior cingulate gyrus

In the present study, it was revealed that the GM volume in the left posterior cingulate gyrus of 19–30-year-old autism group was smaller than that of TD group, and there was no significant difference in the GM volume in the left posterior cingulate gyrus between 6–12-year-old and 13–18-year-old autism group and TD group. Chandley Crawford et al. [71] found that pyramidal neurons in cingulate cortex of autistic adults were lower than those of TD adults, and the gene expression was atypical, which supported the results of the current study. Sussman, Leung [24] studied 72 autistic individuals and 138 TD individuals who aged 4–18 years old, and found that the left cingulate gyrus of autistic individuals gradually thinned with age, while the posterior cingulate gyrus of TD individuals increased with age [58]. This may explain that left posterior cingulate gyrus in this study did not show a significant difference between ASD group and TD group at the ages of 6–12 and 13–18 years old, while the left posterior cingulate gyrus of ASD group was smaller than that of TD group at the age of 19–30 years old. The autopsy study of adult autistic individuals revealed that the cellular structure of posterior cingulate gyrus changed including irregularly distributed neurons, and the boundary between layers IV and V was difficult to distinguish, suggesting that there were atypical patterns of development and migration of neurons in the posterior cingulate gyrus of autistic individuals [72]. Geurts et al. [73] found that the ASD symptom severity was correlated with left posterior cingulate volume, while ADHD symptom severity was associated with the volume of the right parietal lobe. It was suggested that the GM volume in the left posterior cingulate gyrus has important pathological significance for ASD.

Previous studies have shown atypical levels of neurotransmitters in the left posterior cingulate gyrus of autistic individuals, including the decrease of serotonin 5-HT receptors in the autistic adults’ posterior cingulate cortex, and the important role of 5-HT in synaptogenesis, nerve growth, and neuronal migration [74]. Using proton magnetic resonance spectroscopy, Nakamura et al. [75] found that 5-HT decreased in the cingulate gyrus of autistic individuals and was associated with their social cognitive deficits. Levitt et al. found atypical neurometabolites in the left cingulate gyrus of autistic individuals [76].

The cingulate cortex involves various functions, including motor control [77], cognitive control [77], conflict monitoring [78], and social cognition [79]. These functions are partly neuronal functions of cingulate cortex itself and partly functional connections with other brain regions. The cingulate cortex was considered as one of the atypical brain areas that was closely related to the pathology of ASD. Posterior cingulate cortex is a part of the human facial expression processing neural network [80] and an important area of marginal-cortical network that is responsible for social emotional behavior, and is closely associated with social deficits in ASD. A meta-analysis of fMRI studies on ASD found that the activation of cingulate gyrus in autistic adults was significantly weaker than that in TD adults [81]. These findings fully demonstrated the functional atypicalities of the left posterior cingulate gyrus in ASD, which could be due to the local atypical structure of the left posterior cingulate gyrus or the typical connection of neural network in ASD. Another study found that in non-social tasks, the left cingulate gyrus of autistic adults was weaker than that in autistic children [48]. In TD individuals, the functional connectivity between posterior cingulate gyrus and medial prefrontal cortex increased with age, while it decreased with age in ASD group [82]. It is suggested that the left cingulate gyrus and the posterior cingulate gyrus of autistic individuals had different functional levels at different ages, which could be related to the changes of GM volume in the left posterior cingulate gyrus of autistic individuals at different ages.

There are also some inconsistencies between the results of previous results and findings of the current study. For instance, Cauda et al. [83] found that the GM volume in the cingulate gyrus of autistic adult patients increased compared with that of TD adults. A meta-analysis did not find a significant difference in the GM volume of cingulate cortex between autistic adults and TD adults [20]. The heterogeneity among the results of previous studies could be related to the differences in sample size, age, disease severity, and research method [84].

## Conclusions

In conclusion, the current study showed the whole-brain volume, whole-brain WM volume, and whole-brain GM volume of autistic individuals who aged 6–12, 13–18, and 19–30 years old showed no significant difference compared to TD individuals. The brain areas with atypical GM volume of autistic individuals in the three age-based cohorts were different, involving the right superior temporal gyrus, the inferior parietal lobule, the right middle occipital gyrus, and the left posterior cingulate gyrus. These brain areas were of great significance for us to further understand the neuropathological mechanism of ASD. However, the results of the present study were related to only autistic individuals who aged 6–30 years old and cannot be extended to autistic individuals in other age ranges. Additionally, except for the left inferior parietal lobule, other atypical brain areas were only obtained at the uncorrected level (P < 0.001). In the future study, it is essential to expand the sample size and include more autistic individuals with different levels of IQ, and to conduct a rigorous statistical analysis to verify our findings.

## Supplementary Information

**Additional file 1: Table S1. **Whole brain volume comparison between ASD and TD group in Childhood (6-12 years old). **Table S2.** Whole brain volume comparison between ASD and TD group in Adolescents (13-18 years old). **Table S3.** Whole brain volume comparison between ASD and TD group in Adulthood (19-30 years old).

## Data Availability

All the clinical data used to support the findings of this study may be released upon application to the data access manager, who can be contacted at ljyuch@bjmu.edu.cn.

## Abbreviations

ASD: Autism spectrum disorder
sMRI: Structural magnetic resonance imaging
TD: Typically developing individuals
VBM: Voxel-based morphometry
GM: Grey matter
ADOS: Autism diagnostic observation schedule
RRB: Restricted and repetitive behaviors
ADI: Autism diagnostic interview
DSM-IV: Diagnostic and statistical manual-IV
IQ: Intelligence quotient
C-WISC: The Chinese-Wechsler Intelligence Scale for Children
WAIS-RC: The Wechsler Adult Intelligence Scale-Revised in China
TR: Repetition time
TE: Echo time
FOV: Field of view
SPM: Statistical parameter software
WM: White matter
CBF: Cerebrospinal fluid
MNI: Montreal Neurological Institute
FWHM: Full width at half maximum
SNR: Signal-to-noise ratio
