# Supplementary material for: Developmental brain structural atypicalities in autism: a voxel-based morphometry analysis
Source: Child Adolesc Psychiatry Ment Health. 2022 Jan 31;16:7. doi: 10.1186/s13034-022-00443-4 (PMC8805267; doi:10.1186/s13034-022-00443-4)
Supplement: Supplementary file 1 — Additional file 1: Table S1. Whole brain volume comparison between ASD and TD group in Childhood (6-12 years old). Table S2. Whole brain volume comparison between ASD and TD group in Adolescents (13-18 years old). Table S3. Whole brain volume comparison between ASD and TD group in Adulthood (19-30 years old). [file 13034_2022_443_MOESM1_ESM.docx]

**Additional file 1**

**Table S1.** Whole brain volume comparison between ASD and TD group in Childhood (6-12 years old).

|  | ASD  （n=24） | TD  （n=19） | *F* | *P* |
| --- | --- | --- | --- | --- |
| Whole brain volume | 1180.7±123.1 | 1145.2±110.2 | 0.157 | 0.694 |
| Whole grey matter volume | 725.7±77.0 | 699.4±63.7 | 0.315 | 0.578 |
| Whole white matter volume | 454.9±53.2 | 445.8±55.5 | 0.011 | 0.915 |

**Table S2**. Whole brain volume comparison between ASD and TD group in Adolescents (13-18 years old).

|  | ASD  （n=18） | TD  （n=18） | *F* | *P* |
| --- | --- | --- | --- | --- |
| Whole brain volume | 1243.2±95.6 | 1183.0±117.8 | 2.282 | 0.141 |
| Whole grey matter volume | 742.1±66.4 | 698.3±64.2 | 3.076 | 0.089 |
| Whole white matter volume | 501.1±40.1 | 484.7±56.9 | 0.886 | 0.354 |

**Table S3**. Whole brain volume comparison between ASD and TD group in Adulthood (19-30 years old).

|  | ASD  （n=10） | TD  （n=13） | *F* | *P* |
| --- | --- | --- | --- | --- |
| Whole brain volume | 1267.6±130.3 | 1255.8±92.9 | 0.001 | 0.974 |
| Whole grey matter volume | 709.1±80.1 | 715.4±52.0 | 0.523 | 0.479 |
| Whole white matter volume | 558.5±54.9 | 540.4±47.1 | 0.415 | 0.528 |

*Note*: Whole brain volume = Whole grey matter volume + Whole white matter volume
